# Utilizing the sublingual form of squalene in COVID-19 patients: a randomized clinical trial

**DOI:** 10.1038/s41598-024-54843-x

**Published:** 2024-02-24

**Authors:** Mahmoud Ebrahimi, Nafiseh Farhadian, Sara Saffar Soflaei, Alireza Amiri, Davoud Tanbakuchi, Rozita Khatamian Oskooee, Mohammad Karimi

**Affiliations:** 1 Cardiovascular Research Center, Faculty of Medicine, Mashhad University of Medical Sciences, Mashhad, Iran; 2 Chemical Engineering Department, Faculty of Engineering, Ferdowsi University of Mashhad, Mashhad, Iran; 3 Department of Modern Sciences & Technologies, Faculty of Medicine, Mashhad University of Medical Sciences, Mashhad, Iran; 4 Emergency Medicine Department, Golestan University of Medical Sciences, Gorgan, Iran; 5 Department of Biostatistics, School of Health, Mashhad University of Medical Sciences, Mashhad, Iran; 6 Emergency Medicine Department, Birjand University of Medical Sciences, Birjand, Iran

**Keywords:** COVID-19, SARS-COV-2, Squalene, Early treatment, Mortality rate, Re-hospitalization, Immunology, Diseases, Medical research

## Abstract

In this study, the efficacy of sublingual squalene in decreasing the mortality rate among patients with COVID-19 was investigated. Squalene was extracted from pumpkin seed oil with a novel method. Then, the microemulsion form of squalene was prepared for sublingual usage. In the clinical study, among 850 admitted patients, 602 eligible COVID-19 patients were divided in two groups of control (N = 301) and cases (N = 301) between Nov 2021 and Jan 2022. Groups were statistically the same in terms of age, sex, BMI, lymphocyte count on 1^st^ admission day, hypertension, chronic kidney disease, chronic respiratory disease, immunosuppressive disease, and required standard treatments. The treatment group received five drops of sublingual squalene every 4 h for 5 days plus standard treatment, while the control group received only standard treatment. Patients were followed up for 30 days after discharge from the hospital. The sublingual form of squalene in the microemulsion form was associated with a significant decrease in the mortality rate (p < 0.001), in which 285 (94.7%) cases were alive after one month while 245 (81.4%) controls were alive after 1 month of discharge from the hospital. In addition, squalene appears to be effective in preventing re-hospitalization due to COVID-19 (p < 0.001), with 141 of controls (46.8%) versus 58 cases (19.3%). This study suggests sublingual squalene in the microemulsion as an effective drug for reducing mortality and re-hospitalization rates in COVID-19 patients.

**Trial Registration Number:** IRCT20200927048848N3.

## Introduction

The World Health Organization proclaimed a global pandemic of the novel COVID-19 (SARS-Cov-2) in December 2019. It has become a severe worldwide health hazard due to its high infectivity and contagion rate^1,2^. According to recent WHO data, there were 774,075,242 confirmed cases of COVID-19 until January 7, 2024^3^.

COVID-19 infection can range from asymptomatic infection to life-threatening pneumonia and ARDS, cytokine storms, multiple organ failure, and death^4,5^. Growing research proposes that pro-inflammatory cytokines and cells are unusually abundant in critically patients, bringing up the cytokine storm syndrome and hyper-inflammation for COVID-19 etiology^6,7^. On the other hand, oxidative stress is thought to play a role in SARS-Cov-2 infection pathogenesis by contributing to cytokine storm, coagulopathy, and cell hypoxia^8^. Researchers confirmed that the mortality of COVID-19 might be due to inflammation caused by the virus. Continuous positive feedback loops between oxidative stress and pro-inflammatory signaling cause an uncontrolled hyper-inflammation state^9^.

The urgent need for an effective cure has sparked a worldwide competition to discover a unique or repurposed antiviral drug. Several therapeutic drugs are being studied; however, numerous clinical trials are still ongoing. The FDA has only approved Remdesivir for COVID-19 treatment thus far, although the need for therapeutic development persists^10,11^. Additional therapy choices may help patients while reducing the strain on healthcare systems. Although creating innovative therapies is critical, formulating new medications and evaluating their safety and efficacy in a short amount of time appears unrealistic due to the time-consuming nature of the procedure. As a result, it is reasonable to investigate previously used, possible antiviral medicines that are safe and well-tolerated.

Squalene (SQ) has anti-inflammatory^12,13^, immunomodulatory, and antioxidant properties with minimal adverse effects, raising the prospect that it could alter SARS-CoV-2 infection. This natural chemical reduces neutrophil and monocyte activation while increasing anti-inflammatory enzymes^12^. The antiviral effect of squalene in the treatment of the hepatitis C virus is approved in an invention patent^14^. Ulrikh et al. observed that SQ increases the synthesis of anti-inflammatory cytokines, such as IL-4, IL-10, and IL-13, in pro-inflammatory macrophages. Moreover, SQ administration led to remodeling and repair signals, and the recruiting signal of neutrophils and eosinophils is responsible for phagocytosis^15^. Further support for this notion can be found in another in vivo study, which showed that SQ induces an increase in IL-4, IL-10, and IL-13 synthesis and a decrease in pro-inflammatory signals, including NF-κB and TNF-α, in M1 macrophages. Additionally, SQ affects remodeling and repair signals (TIMP-2)^16^.

Moreover, researchers confirmed the anti-oxidative effects of SQ^17,18^, which are very important in viral infection. Antioxidant defense is critical in determining the severity of viral infections^19^. Antioxidant depletion may cause viral mutations and increase viral virulence in RNA viruses^20^. Warleta et al. confirmed that administration of SQ in human mammary epithelial cells (MCF10A) decreases intracellular ROS, suppresses H2O2-induced oxidative damage, and protects DNA against oxidative injury^21^.

Here, the efficacy of SQ in the treatment of COVID-19 patients has been investigated. First, SQ was extracted from pumpkin seed oil with a novel method. After approving the purity of the extracted compound, it was encapsulated in the microemulsion for higher efficacy as a sublingual compound. Then, a clinical study was examined to investigate the efficacy of treatment with SQ while examining the mortality rate in COVID-19 patients. Critical parameters such as the number of hospitalization days of the patients until discharge from the hospital, re-infection with COVID-19, and the type of vaccine were also explored. The main goal and objectives of this study can be summarized as follows:

### Main goal

A clinical study was examined to investigate the efficacy of the sublingual form of SQ in the microemulsion form for COVID-19 patients.

### Main objectives

- Extraction of SQ from pumpkin seed oil with a novel method.
- Encapsulation of SQ in the microemulsion form as a sublingual compound.
- Examining the mortality rate of COVID-19 patients that are treated with this new formulation.
- Exploring the impact of important parameters such as the number of hospitalization days of the patients until discharge from the hospital and re-infection with COVID-19 on treatment efficiency.

## Materials and methods

### Materials

Potassium hydroxide (KOH), FeCl_3_⋅6H_2_O (97%), FeCl_2_⋅4H_2_O (99%), acetone, ethanol, hexane, ammonium hydroxide (27–30%), Tween 80, and Span 80 were purchased from Merck Company (Darmstadt Merck, Germany). Pumpkin seed oil was procured from Zarband Company, Iran. Standard squalene was purchased from Sigma Aldrich Company for Gas Chromatography (GC) analysis.

### Methods

#### Squalene extraction

SQ is naturally widespread in animals, plants, fungi, and bacteria^22^. The primary source of SQ is shark liver oil. SQ extraction from this source is restricted by animal protection regulations^23^. SQ is also extracted from plant sources^24^.

Here, SQ extraction from pumpkin seed oil was performed according to our previous studies^25–27^. In summary, pumpkin seed oil was applied as the main source of SQ extraction with a novel method. At first, fatty acids (FAs) adsorption on the magnetic iron oxide nanoparticles was performed. Then, the solvent extraction method was applied to separate SQ from other components. Briefly, a coprecipitation reaction was used to prepare magnetic nanoparticles (MNPs) in the form of iron oxide (Fe_3_O_4_). Then, the saponification reaction was applied for pumpkin seed oil. Pumpkin seed oil and ethanolic KOH were poured in a container. The container was heated at 80 °C for 1 h. To evaporate all the solvent, the solution was heated in an oven for 4 h. Finally, a soap‐like dried sample was obtained. MNPs were added to this sample that was dissolved in water. This sample was placed in an oven at 130 °C for FAs adsorption on the MNPs surface. Simultaneously, water was removed from this sample. The dried sample was washed with acetone to separate extracted SQ, and MNPs were separated with a magnet. Afterward, hexane, distilled water, and ethanol were added to the remaining solvent and placed in a decanter to separate hexane containing SQ from other materials. Finally, hexane was removed under argon, and SQ was extracted.

#### Microemulsion preparation

For the preparation of the sublingual form of SQ, a microemulsion formulation with the SQ concentration of 10 mg/ml was applied. The titration approach was used to create a microemulsion. For this purpose, a mixture of hydrophilic and hydrophobic nonionic surfactants was used. Surfactant components were mixed in a mass ratio of 9:1 (Tween80:Span80), SQ was added to the surfactant mixture in a mass ratio of 1:5 (SQ: surfactants), and double distilled water was gently added under moderate agitation (magnetic stirring). The final formulation for sublingual usage includes 10 mg SQ, 45 mg Tween 80, 5 mg Span 80, and 1 ml water.

#### Characterization

The purity of extracted squalene was measured using GC‐FID analysis (6890 Series GC system) was applied. A dynamic light scattering analyzer (DLS, CORDOUAN) was used to assess the hydrodynamic diameter of a microemulsion sample in deionized water. TEM (LEO-Germany) analysis was used to assess the morphology and size of the sample. The zeta potential of the microemulsion sample was calculated using a zeta meter (Zeta CAD).

#### Intervention

**Study population** Patients were selected from adults (age > 18 years) admitted to the emergency and infectious department of Shohada Hospital, Ghaen (Iran) between November 2021 and January 2022. Cases with the signs of diagnostic and symptoms of COVID-19, including fever, acute loss of sense and smell, fatigue, dry cough, lymphocytopenia, elevated CRP with or without contact with verified cases of COVID-19, or patients with individual characteristics in HRCT, including ground-glass opacities, bilateral abnormalities, vascular enlargement, lower lobe involvement, and posterior predilection (1), or who confirmed with a real-time PCR test, eligible patients who had respiratory symptoms (including dyspnea, chest pain, or discomfort), oral temperature > 38 °C and SpO_2_ < 93%, have entered the study.

The sample size was calculated according to the findings of the previous study^27^, 300 for each study group, using the following formula (α = 0.01 and β = 0.95):

$${\text{N}} = \left( {{\text{Z}}_{{{1} - \alpha /{2}}} + {\text{Z}}_{{{1} - \beta }} } \right)^{{2}} *{\text{P}}_{{1}} \left( {{1} - {\text{P}}_{{1}} } \right) + {\text{P2}}\left( {{1} - {\text{P}}_{{2}} } \right)/\left( {{\text{P}}_{{1}} - {\text{P}}_{{2}} } \right)^{{2}}$$

Demographic and baseline characteristics, including age, sex, body mass index (BMI), history of diabetes mellitus (DM), hypertension (HTN), IHD (ischemic heart disease), chronic heart disease (CKD), immunosuppressive disease, chronic respiratory disease, the number of vaccination doses, and vaccine type, were recorded.

Exclusion criteria were a history of mental retardation, being directly admitted to the ICU, pregnancy, breastfeeding, a suspected or confirmed history of alcohol or substance use disorder, and having participated in other drug trials in the past month.

After obtaining written informed consent, eligible patients were divided into two groups of standard treatment (control group, N = 301) and SQ plus standard treatment (intervention group, N = 301) using a web-based randomization tool and an allocation concealment mechanism by staff whom were not involved in the study.

**Treatment protocol** Standard treatment included oxygen therapy, dexamethasone 8 mg daily or prednisolone 40 mg daily for 10 days, remdesivir 200 mg for the first day of admission and then 100 mg daily for 5 days, and heparin 5000IU subcutaneous TDS (for BMI ≥ 40, 7500 IU SC TDS) or enoxaparin 40 mg SC once daily (for BMI ≥ 40, 40 mg SC BID). In the case group, standard treatment plus SQ dosage was applied. 5 drops of sublingually SQ was applied every 4 h for up to 5 days.

**Study variables** Vital signs, clinical characteristics, and probable adverse effects of squalene were collected and recorded continuously during hospital admission. Symptom evaluators were blinded.

**Follow-up** Patients were followed up for 30 days after discharge from the hospital in terms of the need for re-hospitalization and mortality rate.

**Figure 1:**
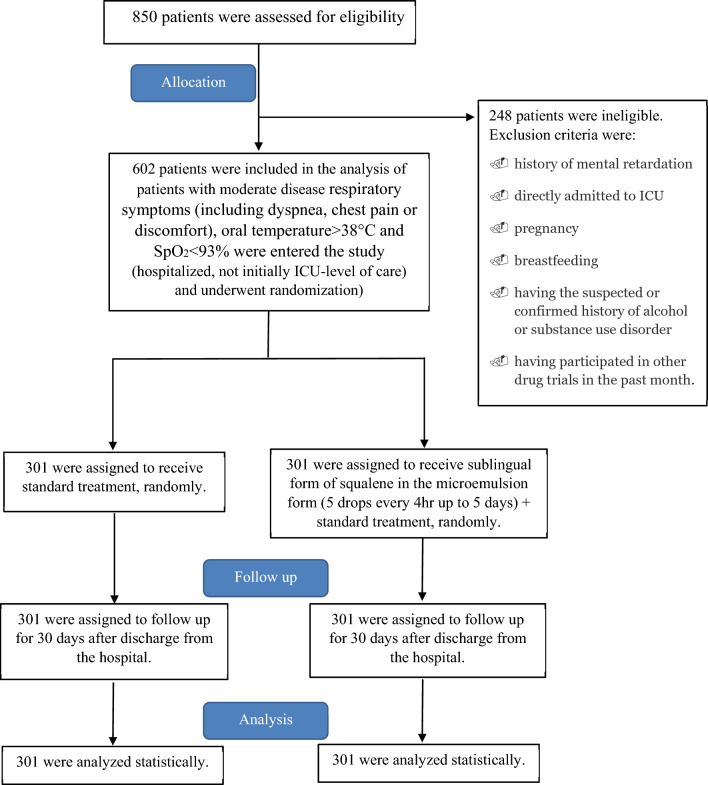
Screening, enrollment, randomization, and clinical protocol.

Figure 1 shows a schematic of patient screening, enrollment, randomization, and clinical protocol that is applied in this study.

### Statistical analysis

SPSS 18 was used to analyze the data. The data analyst was unfamiliar with the study groups. Quantitative factors were expressed as median standard deviation and qualitative variables as number and percent. The Chi-square and T-test was employed to analyze the difference between study groups in terms of baseline characteristics, different types of treatment, and outcomes in the following period. A nonparametric analytical tool (Binary logistic regression) was employed to determine the critical influential factors related to death, re-hospitalization, and duration of hospitalization due to COVID-19. A P value of 0.05 was deemed significant.

### Ethics

The study protocol was approved by the MUMS ethics committee (IR.MUMS.REC.1400.143) and IRCT (IRCT20200927048848N3, date: 10/10/2021). We confirm that all methods were performed in accordance with the relevant guidelines and regulations according to the Helsinki Declaration. Informed consent was obtained from all participants and/or their legal guardians.

### Problems anticipated

Following participants 1 month after admission was our major challenge in this study. For dealing with this problem, we recorded all the participant addresses and also their phone number as well as their cell phone. To confirm the death of participants, we also recorded their national number and checked them in the death registry database.

### Limitations of the study

One of the limitations of this study was the presence of intervening factors such as age, co-morbidities of the patients and patients’ personal medications, which could influence the relationship between SQ use and death. Another limitation of this study was the impossibility of subgroup analysis for all outcomes due to time constraints and a lack of research manpower (this research was conducted during the peak of the Iran’s COVID-19 crisis), so only the outcome of the need for oxygen therapy (a clinical outcome) was subjected to subgroup analysis. As a result, subgroup analysis was not performed based on characteristics such as SQ treatment duration and prescription dose. Furthermore, conducting the study in one center and the possibility of recall error were other limitations of this study.

However, critically ill COVID-19 patients had lower chance of receiving SQ treatment during hospitalization, this bias could account for the link between not utilizing SQ and poor outcomes.

### Duration of the project

Squalene extraction and characterization: September–October 2021 (2 months).

Clinical trial sampling and following-up: November 2021-January 2022 (3 months).

Data entry and statistical analysis: February–March 2022 (2 months).

## Results

### SQ extraction

**Figure 2:**
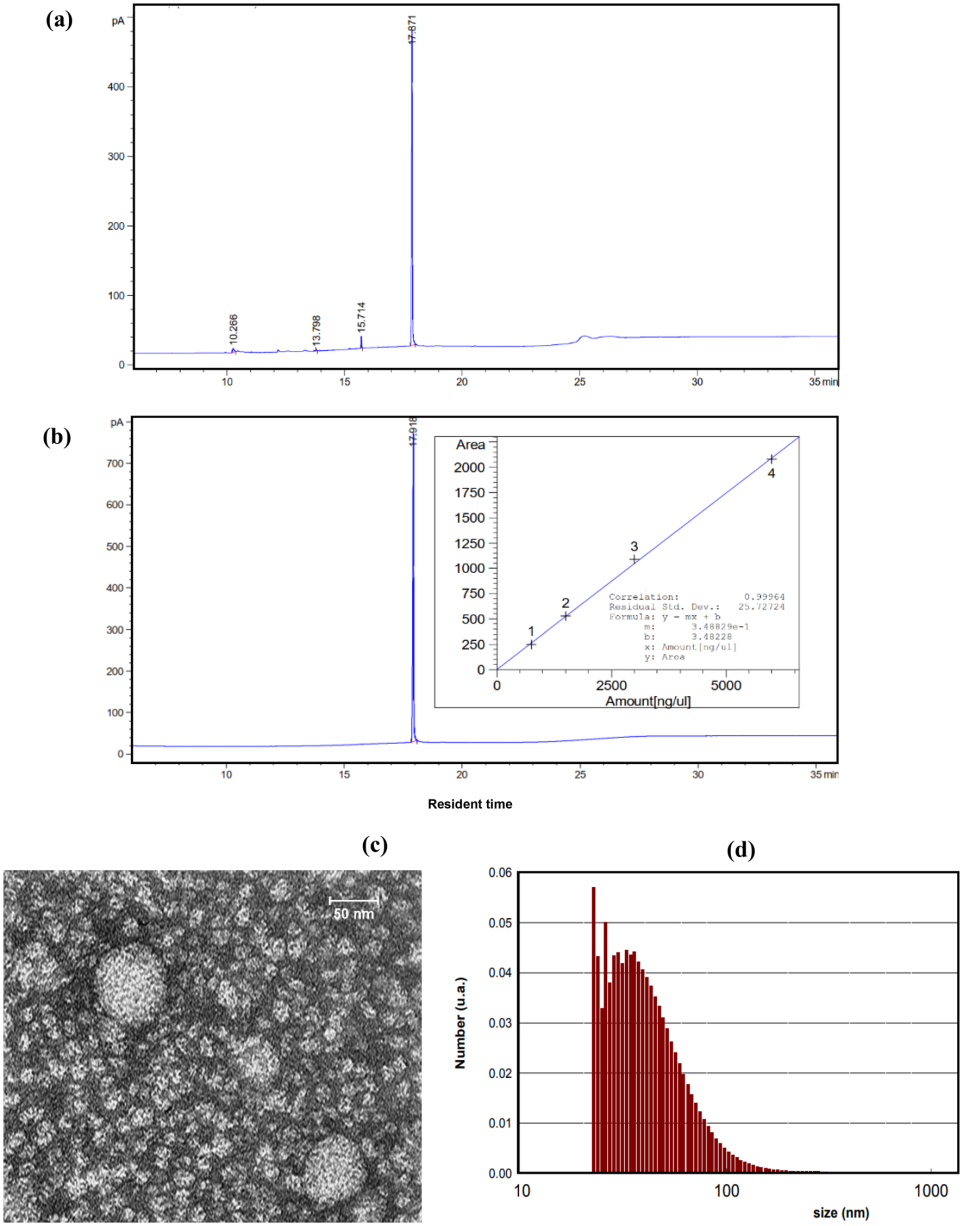
(**a**) FID‐GC chromatogram diagram for extracted squalene at 3250 ppm, (**b**) FID‐GC chromatogram diagram for standard squalene at 6000 ppm (incident diagram shows the calibration curve), (**c**) TEM image, and (**d**) DLS diagram, of the sublingual form of SQ in microemulsion form.

SQ purity was detected using FID‐GC analysis. Results confirmed that SQ purity was higher than 95%. Figure 2-a,b show the FID‐GC diagram of extracted and standard SQ with the calibration curve. After approving the purity of SQ, the microemulsion form of this component was prepared for sublingual usage. Figure 2-c,d show the size diagram versus TEM and DLS analysis, respectively. As Fig. 2-c illustrates, the sample contains spherical particles with an average particle size of about 30 nm. In addition, an average particle size of 40.37 nm with a polydispersity index (PDI) of 0.247 is shown in the DLS diagram (Fig. 2-d). The zeta potential of the microemulsion sample was calculated as − 32.01 mV. The pH value of the prepared microemulsion containing SQ was 6.52, which is safe for the sublingual usage.

### Study population characteristics

Total of 602 COVID-19-confirmed patients were allocated into SQ plus standard treatment (case, N = 301) and the standard treatment (control, N = 301) groups. The average age in cases was 58.4 ± 0.49 years, and it was 59.02 ± 0.45 years in the control group.

Regarding baseline characteristics (Table 1), there was no difference between case and control groups in age (p = 0.352), sex (p = 0.552), BMI (p = 0.600), 1st day of admission lymphocyte count (p = 0.197), history of DM (p = 0.930), history of IHD (p = 0.782), history of HTN (p = 1.000), history of CKD (p = 0.887), history of chronic respiratory disease (p = 0.870), history of immunosuppressive disease (p = 1.000), one dose vaccination (p = 0.791), two dose vaccination (p = 0.623) and vaccine type (p = 0.798).

**Table 1:** The comparison of demographic and past medical history between the studied groups.

Variable	Case (N = 301)	Control (N = 301)	P value
Age (years)	58.40 ± 0.49	59.02 ± 0.45	0.352
Sex n (%)			
Male	190 (63.12%)	197 (65.45%)	0.552
Female	111 (36.88%)	104 (34.55%)	
BMI (kg/m^2^)	28.51 ± 0.09	28.52 ± 0.09	0.600
1st day lymphocyte count (n/µl)	917.34 ± 190.34	927.63 ± 203.89	0.197
HTN n (%)			
Yes	161 (53.49%)	161 (53.49%)	1.000
No	140 (46.51%)	140 (46.51%)	
DM n (%)			
Yes	92 (30.56%)	93 (30.9%)	0.930
No	209 (69.44%)	208 (69.1%)	
IHD n (%)			
Yes	48 (15.95%)	48 (15.95%)	0.782
No	253 (84.05%)	253 (84.05%)	
CKD n (%)			
Yes	22 (7.3%)	23 (7.6%)	0.877
No	279 (92.7%)	278 (92.4%)	
Chronic respiratory disease n (%)			
Yes	137 (45.51%)	139 (46.18%)	0.870
No	164 (54.49%)	162 (53.82%)	
Immunosuppressive disease n (%)			
Yes	17 (5.65%)	17 (5.65%)	1.000
No	284 (94.35%)	284 (94.35%)	
One dose vaccination n (%)			
Yes	207 (68.77%)	210 (69.77%)	0.791
No	94 (31.23%)	91 (30.23%)	
Two dose vaccination n (%)			
Yes	130 (43.19%)	136 (45.18%)	0.623
No	171 (56.81%)	165 (54.82%)	
Vaccine type n (%)			
0 (None)	94 (31.23%)	91 (30.23%)	0.798
1 (Sinopharm)	94 (31.23%)	83 (27.57%)	
2 (Astrazeneca)	53 (17.61%)	63 (20.93%)	
3 (CovIranBarakat)	27 (8.97%)	44 (14.62%)	
4 (Other)	33 (10.96%)	20 (6.64%)	

*BMI* body mass index, *HTN* hypertension, *DM* diabetes mellitus, *IHD* Ischemic heart disease, *CKD* chronic kidney disease.

In addition, there was not any significant difference between two groups in terms of receiving further treatment, including remdesivir (p = 1.000), glucocorticoid (p = 0.886), O_2_ therapy with a low-flow nasal cannula or face mask (0.858), and O_2_ therapy through noninvasive mechanical ventilation (p = 0.929). Table 2 shows details of the comparison between the studied groups.

**Table 2:** Comparison of other treatments between the studied groups.

Variables	Intervention (N = 301)	Control (N = 301)	P value
Remdesivir n (%)			
Yes	245 (81.4%)	245 (81.4%)	1.000
No	56 (18.6%)	56 (18.6%)	
Glucocorticoid n (%)			
Yes	274 (91.03%)	275 (91.03%)	0.886
No	27 (8.97%)	26 (8.97%)	
Low-flow nasal cannula or face mask n (%)			
Yes	211 (70.1%)	213 (70.76%)	0.858
No	90 (29.9%)	88 (29.23%)	
Noninvasive mechanical ventilation n (%)			
Yes	90 (29.9%)	91 (30.23%)	0.929
No	211 (70.1%)	210 (69.77%)	

### Prediction of SQ treatment

The sublingual form of squalene in the microemulsion form was associated with a significant decrease in the mortality rate, in which 94.7% of patients were alive after one month, while the percentage of alive patients was only 81.4% in the control group.

**Figure 3:**
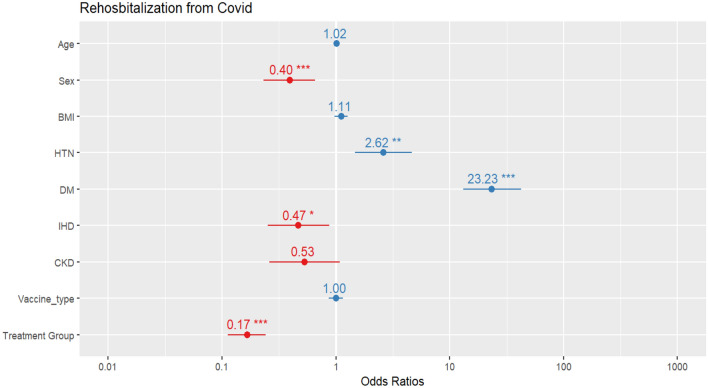
The risk of re-hospitalization due to COVID-19 for each variable (*p-value between 0.01 and 0.05, **p-value between 0.05 and 0.001, and ***p-value less than 0.001).

Figure 3 shows the estimated values of the chance ratio of re-hospitalization for each variable. According to this graph, having diabetes dramatically increases the risk of being re-hospitalized due to COVID-19 by 23 times. Gender in terms of being male, suffering from high blood pressure, and having ischemic heart disease also increases the risk of being re-hospitalized due to COVID-19. SQ plus standard treatment has a significant effect on preventing re-hospitalization, up to 17% less compared to the standard treatment.

**Figure 4:**
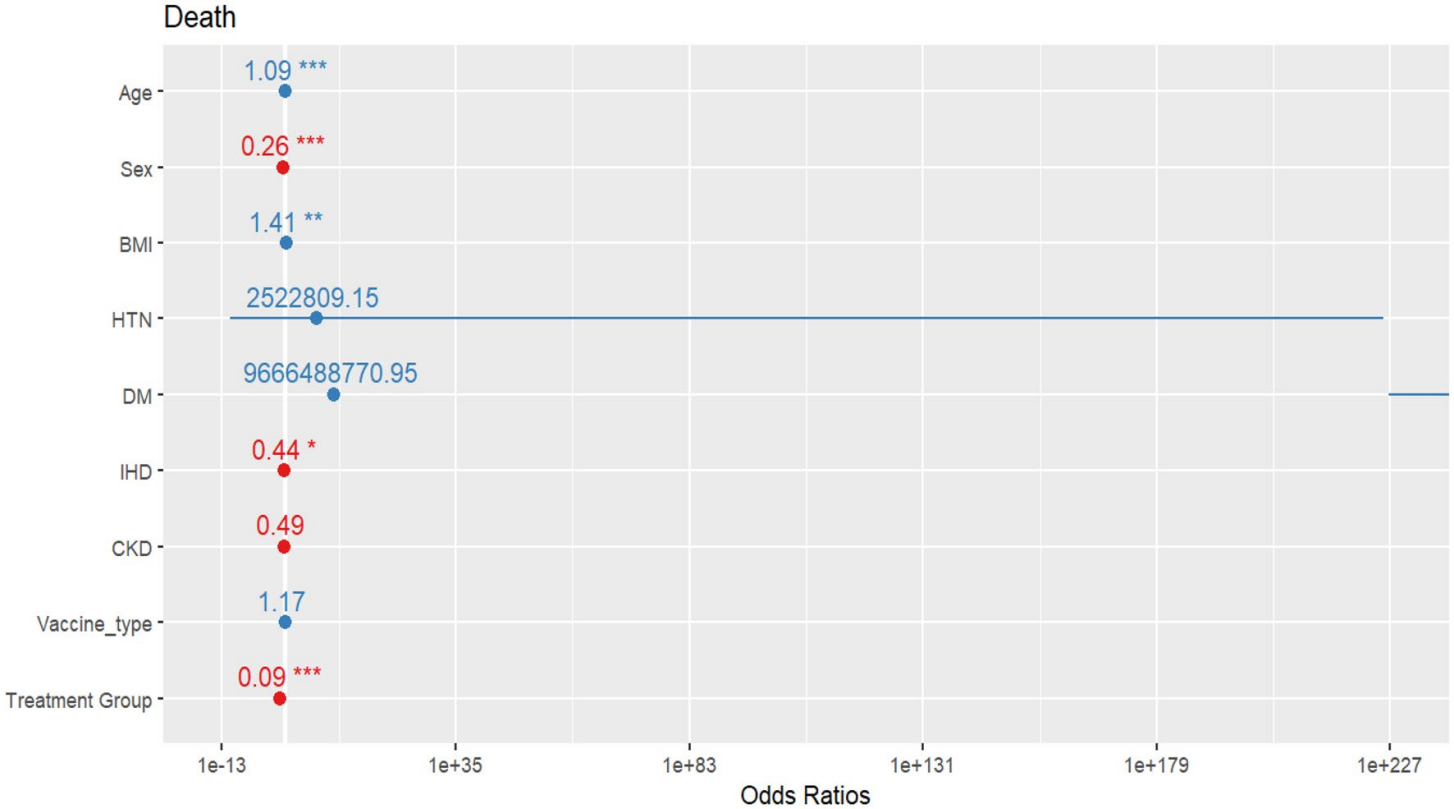
The risk of death for each variable (*p-value between 0.01 and 0.05, **p-value between 0.05 and 0.001, and ***p-value less than 0.001).

Figure 4 shows the estimated odds ratio of the mortality rate for each variable. The sign of * indicates the significance of the variable impact on the probability of patients’ death due to infection with COVID-19. Results show that sex, age, body mass index, suffering from ischemic heart disease, and treatment with SQ affect the probability of death. Also, SQ plus standard treatment reduces the chance of death due to COVID-19 by 9% compared to the standard treatment.

**Figure 5:**
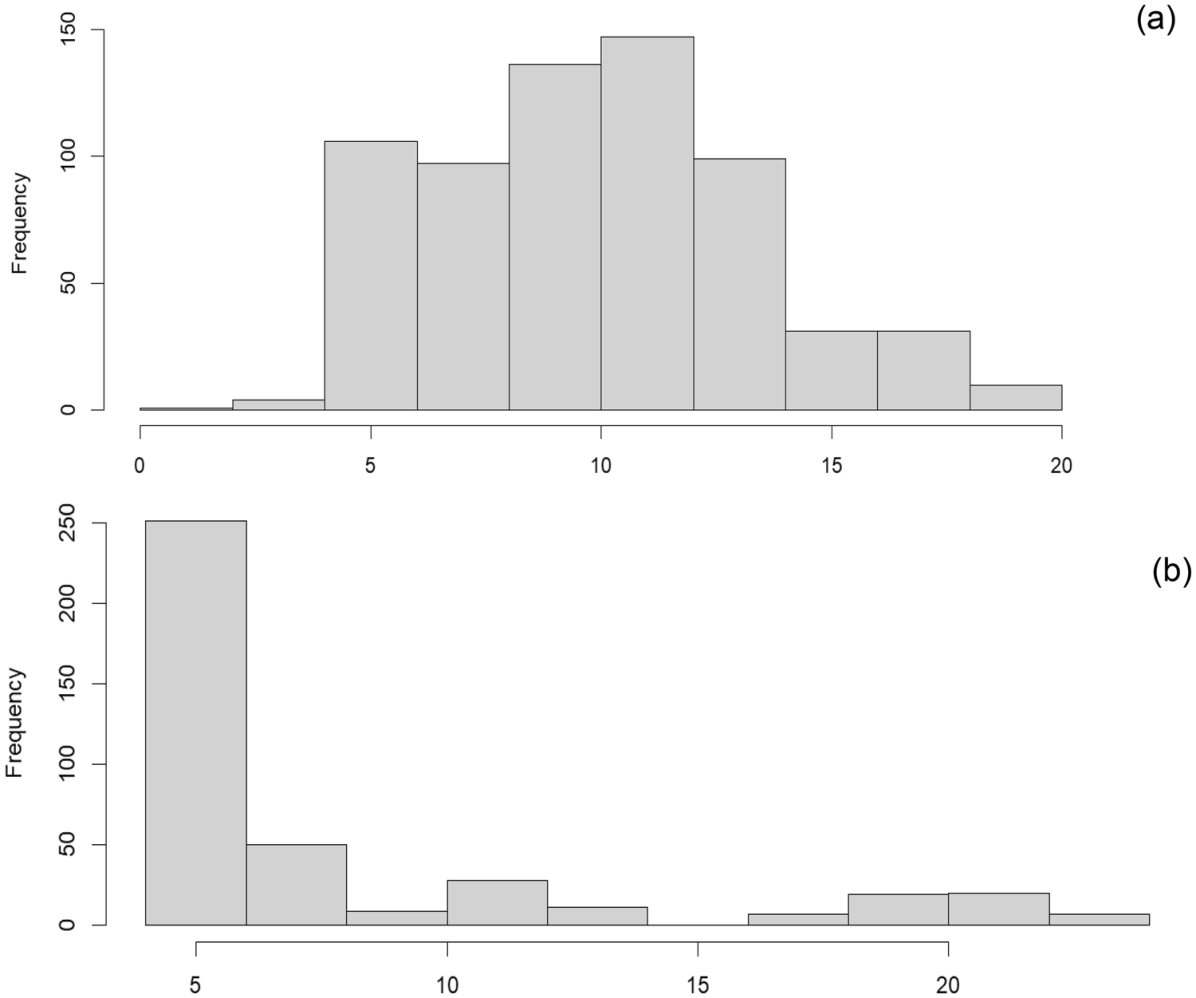
Histogram of hospital discharge for (**a**) the standard treatment group and (**b**) the SQ plus standard treatment group.

Figure 5 shows a comparison between the number of hospitalization days for two treatment groups. These histograms show that the number of hospitalization days of the patients until discharge from the hospital is less in the SQ plus standard treatment group compared to the standard treatment group.

**Figure 6:**
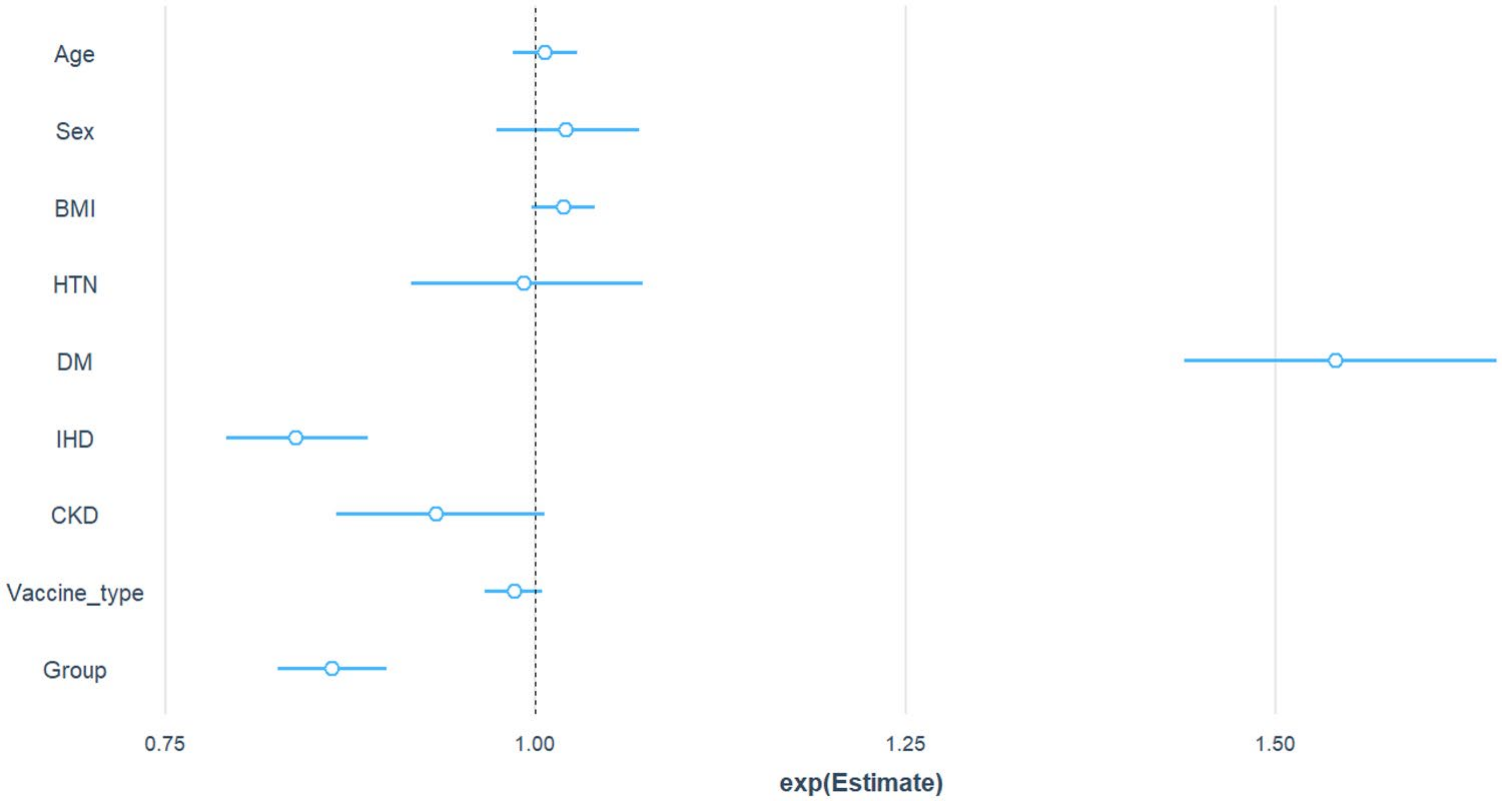
Impact of various parameters on the number of hospitalization days of COVID-19 patients.

Furthermore, our findings show that SQ plus standard treatment increases the chance of a patient's discharge from the hospital one day earlier by 86% compared to the standard treatment group. Diabetes and ischemic heart diseases also have an effect on the number of days of hospitalization for COVID-19 patients in the hospital. In addition, the type of vaccine received does not have a significant effect on the mortality, re-infection, and number of days of hospitalization of patients with COVID-19 (Fig. 6).

## Discussion

In the current clinical trial, we investigated the efficacy of SQ sublingual drops in the treatment of COVID-19. Our data showed that this novel medicine is associated with fewer deaths in COVID-19 patients. We previously indicated, for the first time, the therapeutic effect of SQ microemulsion in SARS-Cov-2 in a clinical trial^27^.

Recently, researchers confirmed that mortality in COVID-19 might be due to inflammation caused by the virus. Continuous positive feedback loops between oxidative stress and pro-inflammatory signaling cause an uncontrolled hyper-inflammation state^9^.

In addition, as mentioned earlier, the NF-κB pathway appears to be a critical factor in COVID-19 natural development and conversion to a severe phenotype^28^. Blocking the NF-κB pathway may lower mortality in the severe type of COVID-19^28–30^. Previous findings confirmed that SQ induces a decrease in pro-inflammatory signals, including NF-κB, and TNF-α in M1 macrophages. However, it can lower the mortality rate of COVID-19 patients^16^. Furthermore, based on the previous studies, it was indicated that the count of CD8+ T cells, T regulatory cells and memory CD4+ T cells had been significantly reduced during COVID-19^31^. It has been reported that in mice, SQ-based emulsion enhances CpG-mediated augmentation of CD8 (+) T-cell responses^32^. In addition, it was shown that SQ enhances the differentiation of monocytes to dendritic cells, as well as the stimulation of antigen-specific CD4 T cell response^33^. Furthermore, in a recent study that applied lipid SQ nanoparticles (SQ@NP) as an adjuvanted COVID-19 vaccines in an animal model, the secretion of cytokines in splenocytes, such as interferon (IFN)-γ, interleukin (IL)-5 and IL-10, was significantly enhanced after adjuvanted of S-protein vaccine with SQ@NP. This research suggested that SQ@NP may be a potential tool for reinforcing T-cell immunity^34^. In another study, the immunogenicity of the Spike RBD of SARS-CoV-2 formulated with an oil in water emulsion and a water-in-oil emulsion with SQ was evaluated in mice and hamsters. Results of immunization assays showed that the formulation based in water-in-oil emulsion of SQ generated an earlier humoral response. Moreover, this formulation was able to stimulate neutralizing antibodies in hamsters. Results confirmed that SQ base emulsion was safe, as demonstrated by the histopathological analysis in lungs, liver, and kidney^35^. Due to the effective properties of SQ, Solomadin et al., applied SQ in NARUVAX-C19 vaccine as an emulsion adjuvant and obtained robust broadly neutralizing humoral and T cell IFN-γ responses^36^. Based on these findings, the role of SQ as an anti-inflammatory, immune-modulatory, and antioxidant drug offers promise in the treatment of COVID-19 patients.

According to previous studies that used SQ adjuvanted vaccines, there was not elicitation of any antibodies against SQ^37^. However, some side effects attributed to vaccines containing SQ adjuvant include narcolepsy, hematologic complications, immunologic complications, complications related to drug formulations, cardiovascular complications and the development of autoimmune diseases (potentially autoimmune origin). These side effects are extremely rare, and according to the literatures, the cause-and-effect relationship between SQ use and these side effects has not been established^38^.

It should be emphasized that no immunological thrombocytopenic purpura and immunologic reaction (hypersensitivity reaction and anaphylaxis) following SQ therapy were reported in this investigation.

However, studied population included only hospitalized participants; therefore, generalizing the findings to the entire population with COVID-19 problems in a non-hospital setting requires caution.

## Conclusion

In the current clinical trial, the efficacy of SQ sublingual drops in the treatment of COVID-19 patients was investigated. Our findings showed that sex, in terms of being male, suffering from high blood pressure, and having ischemic heart disease, increases the chance of being re-hospitalized due to COVID-19. SQ plus standard treatment has a significant effect on preventing re-infection with COVID-19, decreasing the mortality rate and the number of hospitalization days of the patients until discharge from the hospital compared to standard treatment. No adverse effects were observed during the intervention in COVID-19 patients. The anti-inflammatory, immune-modulatory, and antioxidant properties of SQ are responsible for its effectiveness in the treatment of COVID-19 patients. In line with our previous clinical trial on the effect of a micro-emulsion of squalene extracted from pumpkin seed oil, we indicated that this extraction in the form of a sublingual drop could significantly decrease the death rate due to COVID-19. It should be underlined that further studies are needed to investigate the safety of combining SQ with liver antiviral drugs or combining SQ with steroids and ARB/ACEi therapies as well as standard protocols for treating COVID-19 patients. However, a comprehensive study in Iranian society by assuming all possible influential characteristics is recommended. Future research could be more explicitly stated, especially regarding long-term effects and potential applications in different stages of COVID-19.

## Data Availability

The data that support the findings of this study are available from the corresponding author, NF, upon reasonable request.
